# Challenges of access to medicine and the responsibility of pharmaceutical companies: a legal perspective

**DOI:** 10.1186/s40199-016-0151-z

**Published:** 2016-05-04

**Authors:** Saeed Ahmadiani, Shekoufeh Nikfar

**Affiliations:** Department of Pharmacoeconomics and Pharmaceutical Administration, Faculty of Pharmacy, Tehran University of Medical Sciences, 16Azar St, Tehran, Iran

**Keywords:** Access to medicine, Pharmaceuticals, Pharmaceutical companies, Human rights

## Abstract

The right to health as a basic human right- and access to medicine as a part of it- have been a matter of attention for several decades. Also the responsibilities of different parties- particularly pharmaceutical companies- in realization of this right has been emphasized by World Health Organization. This is while many companies find no incentive for research and development of medicines related to rare diseases. Also some legal structures such as “patent agreements” clearly cause huge difficulties for access to medicine in many countries. High prices of brand medicine and no legal production of generics can increase the catastrophic costs- as well as morbidity-mortality of medication in lower income countries. Here we evidently review the current challenges in access to medicine and critically assess its legal roots. How societies/governors can make the pharmaceutical companies responsible is also discussed to have a look on possible future and actions that policy makers- in local or global level- can take.

## Background

Responsibilities of pharmaceutical companies with regard to human rights have been matter of debates for many years. In August 2008, the Secretary-General of United Nations published a report which mentioned that over 2 billion people all over the world do not have sufficient access to essential medicine [1]. The message was clear, two billion people (about one third of the world population at the time) were in danger of death or major harm to their health as a result of the lack of access to essential medicines, either because of not enough attention from pharmaceutical companies, or because the state parties could not fulfill their obligation in regards to essential medicines.

Now after a couple of years it might be still a question that, what the responsibilities of different parties (such as pharmaceutical companies, governments, NGOs, world organizations etc.) are for solving this problem, and how we can assure that the realization of access to essential medicines takes place? This paper will discuss these questions briefly from a human rights perspective, and we will try to find and summarize some legal solutions for controversies and complexities in this field.

## What are the roots of the problem?

Huge part of barriers in access to medicine returns to patent law and its consequences. Although patent law generally has been used for centuries [2], the manifestation of TRIPS agreement in 1994 turned it to a new form of challenge. This agreement force the World Trade Organization (WTO) members to take action for protecting intellectual property rights, which entails that any patented product should be produced, imported, sold or used under permission of the patent owner [3]. This includes medicine, thus the production of each medicine is initiated with a period of monopoly in the market with the highest possible price. In this period there will be no low price generic drugs in the market after signing the agreement by one state (for those drugs which are still under patent), and hence, patients should provide the expensive branded medicine either out of pocket or by using their insurance.

**Fig. 1:**
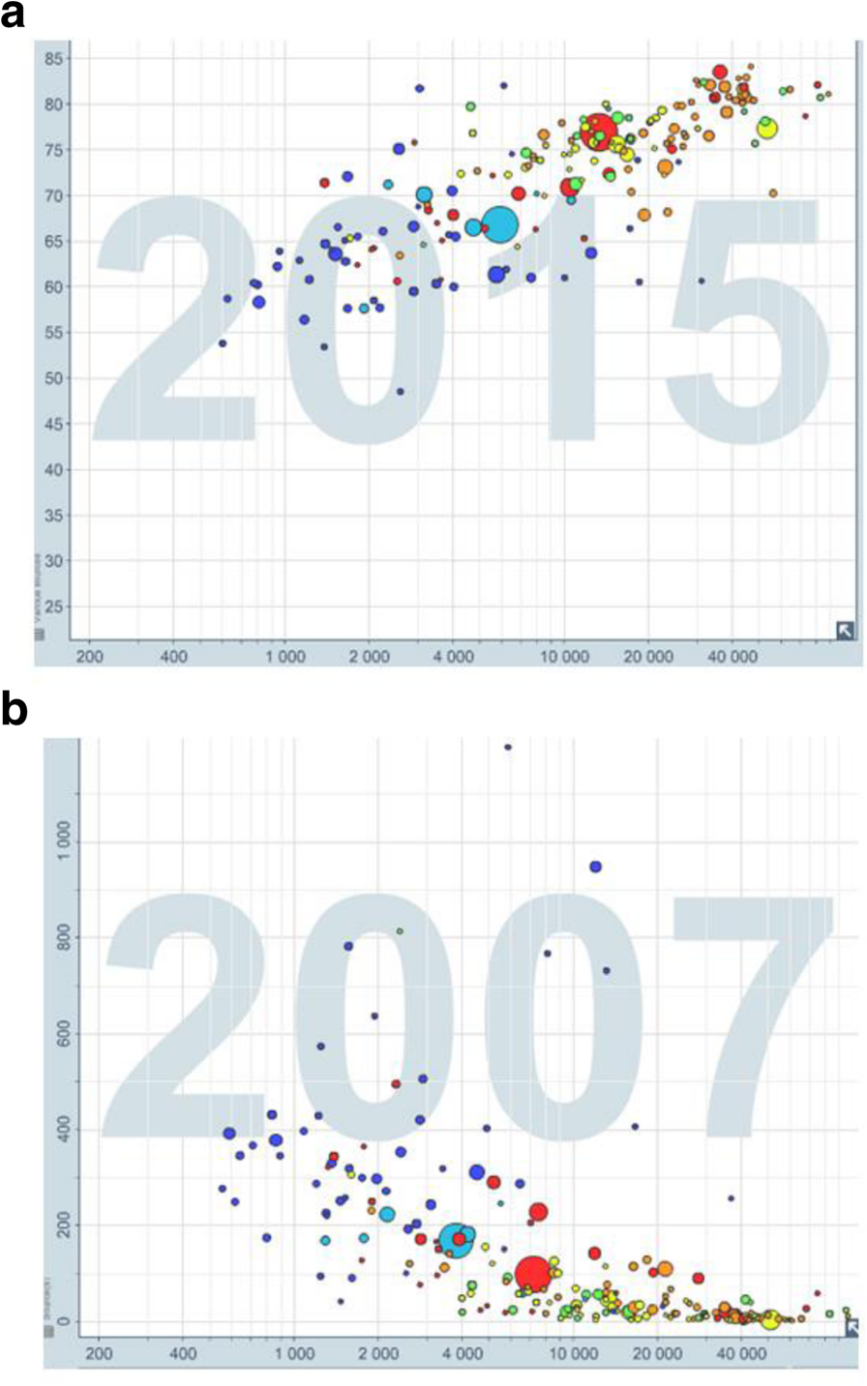
Health and wealth relationship: **a** Increase in life expectancy by wealth; wealthier countries (higher GDP per capita) having higher life expectancy, showing a health inequality in the world; **b** higher prevalence of TB- as a chronic communicable disease- in low income countries. Free material from www.gapminder.org

**Fig. 2:**
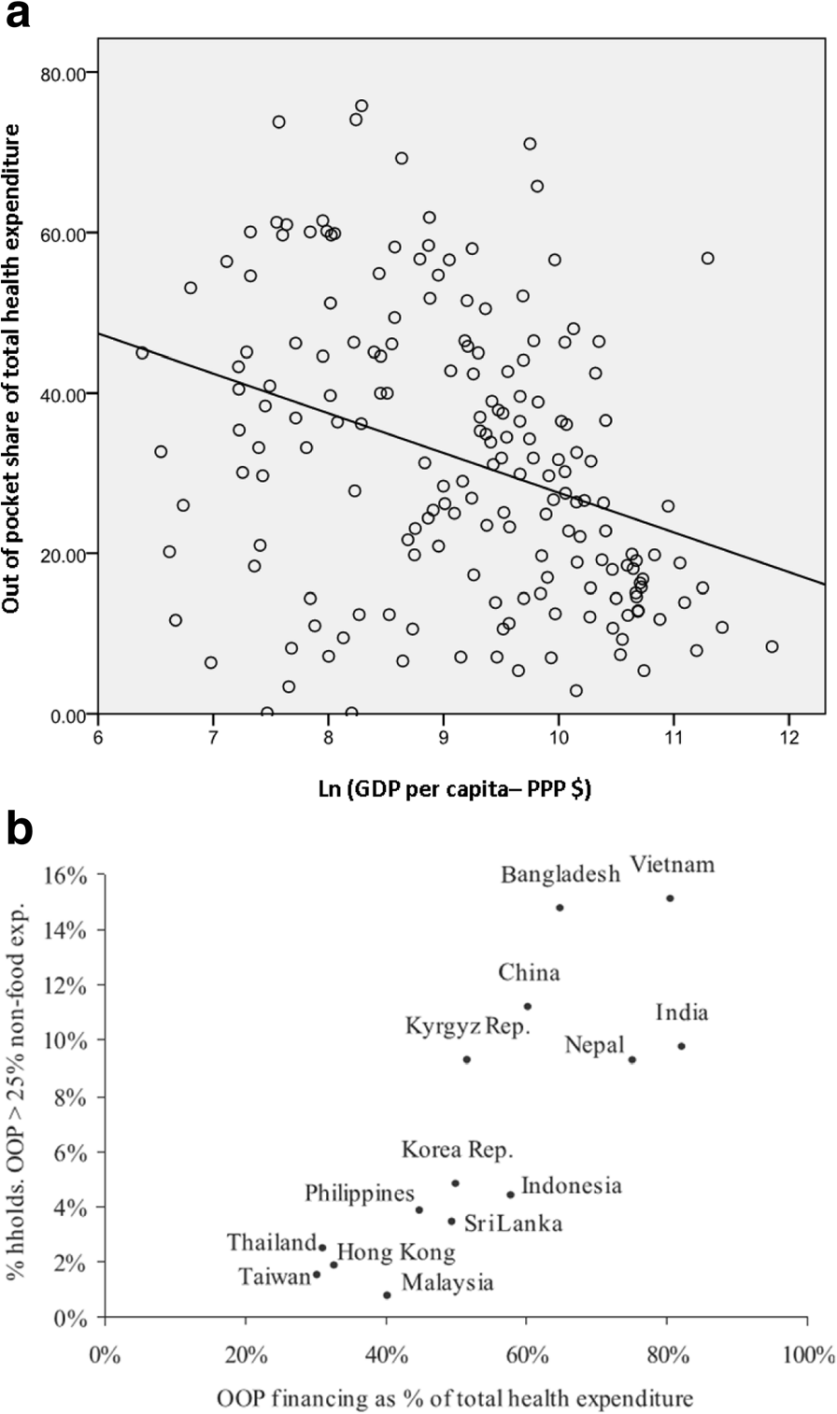
Lower health insurance services in lower income countries: **a** decrease in out-of-pocket (OOP) share of health expenditures by increase in GDP per capita (Data from World Bank [21, 22]); **b** increase in catastrophic expenditures by increase of OOP share; plot from Van Doorslaer et al. [7] (with copy right permission from John Wiley and Sons). These figures depict that lower income can result in lower health insurance services (higher out-of-pocket expenditure for health) and hence higher incidence of catastrophic expenditures consequently, which can cause an inequality in health and social gaps between populations

The problem will rise up when it comes to a developing country where population not only have lower economic status, but also lower health status and higher needs to medicine. According to WHO, life expectancy in developed countries was 1.7 fold higher than developing countries in 2002, showing a 32-years gap in life expectancy between these societies [4]. Also, data shows that infectious diseases such as TB have a negative relationship with GDP per capita of the country [5] (also see Fig 1). These health measures make it obvious that in developing countries there is a higher need to medical technologies which many of them are under patent. At the same time, health insurance coverage is usually poor in these countries and patients often have to pay for the branded medicine out of their own pockets. Evidence shows that the lower the national income is, the higher the out of pocket share of health spending will be [6]. With higher needs and lower economic ability, providing branded medicine will result in a large load of expenditure for states, catastrophic expenditures for patients [7] and increase of mortality and/or morbidity because of low access to medicine (see Fig 2).

Moreover, if any TRIPS member produce or provide an under patent product, the company can sue the member state and ask for a fine compensating the market loss. This was the case for South Africa in late 90s, when giant pharmaceutical companies such as GlaxoSmithKline filed a lawsuit to the Pretoria High Court against the South African government because of importation of generic anti-retroviral medicine- for treating HIV/AIDS endemic situation [8]. The Pharmaceutical Association was using this law to save their presence in the pharmaceutical market of South Africa. However, there were millions of people suffering from HIV/AIDS while could not afford the original brand medicine and the South African state was trying to find a way to guarantee their health. After three years of clashes, the court overruled the patent law in the case and recognized the right to health as a basic human right for the South African patients. Consequently big pharma companies withdrew the lawsuit and started negotiations for dropping the price of original brand to come into the South Africa market [9].

Although this was a happy-ending experience, no country can be sure that the court will give the right to the member state again and hence, in many cases the government prefer to import the branded medicine from the beginning, even if it is not affordable for a part of population.

The TRIPS agreement is not the end of story. Less than a decade after the first TRIPS agreement, United States started to make bilateral trade agreements with other TRIPS members to expand and deepen the TRIPS agreement. These agreements (generally known as TRIPS-plus) decrease the flexibilities which were anticipated for some exceptional situations- particularly for developing countries- and increase the duration of patents in some cases. Until now, there are 20 countries that accepted such an agreement with US [10], which surprisingly 80 % of them are developing countries. If we consider the economic power of United States and its role in pharmaceutical industry, then it is not hard to guess about the effect of these agreements on the access to medicine in the subjected developing countries.

Besides the patent law and TRIPS-plus agreements, there is always a bias towards maintenance medicine- the controlling medicine for chronic conditions. Pharmaceutical companies have a substantial desire in developing drugs which are focused on disease areas within the developed world, such as chronic diseases and cancer treatments, not only because of high prevalence, but also because these drugs are often used in long term, which means a long term costumer for the company, particularly if one can take the advantage of patent. As an instance, a new anti-hypertensive medicine not only has more costumers, also most of the costumers have to use the medicine until the end of their lives, let’s say 10–15 years in average. On the other side, giant pharmaceutical companies are less interested in modern anti-parasites, antibiotics and other medicines related to acute conditions, while these medicines are more needed in developing countries and this bias cause a lower access to medicine- and a lower health in result- in these low income areas.

The mentioned bias also can be seen against “rare diseases” (i.e., diseases with prevalence less than 1/2000), even if they might be chronic. This inattentiveness to some specific diseases forms when the disease is rare or restricted to some particular areas and population, hence pharmaceutical companies find no incentive to invest on research and development of new medicine specified for a limited population, specifically when there is a large possibility that the state does not have the ability to pay for the medicine and the company should provide it underprice.

To see it evidently, from over 1500 drugs which have been approved during 1975–2004, only about 1 % of them were related to the diseases which are known as neglected [11], while over 10 % of global burden of disease is caused by these diseases [12]. This is also reflected in 10/90 phenomena: only 10 % of R&D expenditures is related to problems of 90 % of world population [13]. These facts clearly show an insufficient attention from pharmaceutical companies to this field of health needs. According to WHO, already over one billion people are affected by neglected tropical diseases [14], which may considerably decrease both the life expectancy and quality of life. By considering the higher rate of these diseases in low income countries, it is to say that this situation can cause a huge discrimination between high and low income societies, not only in terms of health, but also economically as a consequence of low health level.

All these modern structures, from patents and TRIPS-plus agreements to bias in pharma industry, cause a decrease or imbalance in access to medicine, and hence an inequity in health between and within the communities, which can be considered as a breach of human rights as will be explained further.

## Human rights and the role of pharmaceutical industry

Previously in 2000, United Nations established eight goals as Millennium Development Goals and 190 countries agreed to help to achieve these goals by 2015. At least three of these goals- reduction of child mortality, improve of maternal health, and combating HIV/AIDS, malaria, and other diseases- are extremely dependent on accessible and affordable medicine. Even the role of pharmaceuticals is clearly mentioned in the millennium declaration: “Develop a global partnership for development- In co-operation with pharmaceutical companies, provide access to affordable, essential drugs in developing countries; proportion of population with access to affordable essential drugs on a sustainable basis” [15].

The strong influence of pharmaceutical companies on accessibility and affordability of medicine is clear. But should they be responsible for the realization of access to medicines?

As first point, health is considered as a basic human right, as it is stated in article 12 of International Covenant on Economic, Social and Cultural Rights, “the States Parties to the present Covenant recognize the right of everyone to the enjoyment of the highest attainable standard of physical and mental health” [16]. And we should beware that, according to UN, the responsibilities which are stated in human rights declaration are not solely an obligation for the member states, but the private sector is equally subjected to human rights responsibilities [17].

Moreover, investing on modern chronic disease and neglecting other diseases such as tropical infections (e.g., leishmaniasis), is clearly inequitable and can increase the rich-poor gap. This socioeconomic gap is an issue to be noticed and should be prevented, which is not possible without serious contribution of pharmaceutical companies. One may mention the super costly research and development in the field of rare disease, but in response we should look at the both sides; costs and revenues. Although it is said that pharmaceutical companies spend around $60 billion for research and development, it should be also mentioned that the annual revenue of these companies exceeds 300 billion [18], which easily covers the cost of R&D. With such an income, it is just an insincere gesture to talk about the R&D costs as a reason of keeping high price for the products.

But even with accepting all these responsibilities, what or who can make the pharmaceutical companies responsible and to make commitment to human rights?

## Making big pharma responsible: legal and social capacities

Since start of these debates, there have been several suggestions for achieving accountability of pharmaceutical companies. These suggestions are mainly classified in two groups of top-down (interventions from governments and international regulators) and bottom-up (pressure from NGOs and social organizations).

## Top-down

As mentioned before, highest attainable standard of health, which includes access to essential medicines, is one of the human rights that states should ensure. Legally, governments that have signed the international treaties of human rights are expected to prevent violation of human rights even. Legislative bodies can play a major role in steering the activities of big pharma and prevent such violations. At first glance, governments might not be able to restrict the pricing directly and/or their trade activities since it is against WTO regulations and TRIPS agreement. Moreover, it is too complicated to define a definite crime in this field, in a way that no one can escape from the law, and at the same time not making barriers for the enjoyment of the right of free trading. But in the wide field of pharmaceutical trading, there are still other ways to make appropriate restrictions, directly and indirectly.

Currently the most applicable way is antitrust or competition law, which protect the presence of competition in the market and fight against corruptions. Many patent owner may find it tempting to have the monopoly of market and be the only brand even after patent expiration. Hence these companies may form secret agreements with generic producers to postpone the entrance of low price generic medicine into the market. Antitrust law bans any act that big pharma companies can take to stop other companies from producing the generic products after patent expiration. By saving the competition, this law guarantee the decrease of price in a reasonable time and hence, increase of affordability of the medicine. This way can be very effective for medicines which their patent is already expired or is going to expire in near future.

Another important step to solve the patent problem was taken by WTO itself with Doha Declaration in 2001. After all debates and discussions on how TRIPS agreement can harm human population health, finally member states decided to make an escape way for emergency situations by waiving the strict TRIPS agreement: “the gravity of the public health problems afflicting many developing and least-developed countries, especially those resulting from HIV/AIDS, tuberculosis, malaria and other epidemic… the TRIPS Agreement does not and should not prevent Members from taking measures to protect public health” [19]. It also clearly mentions the assuming public health problems in paragraph 5 (c): “Each Member has the right to determine what constitutes a national emergency or other circumstances of extreme urgency, it being understood that public health crises, including those relating to HIV/AIDS, tuberculosis, malaria and other epidemics, can represent a national emergency or other circumstances of extreme urgency” [19]. Unfortunately these efforts were not much of success and major countries such as United States, Japan, Switzerland and Australia did not make any domestic legislative implementation for TRIPS Waiver while over 100 countries had accepted to do so [20].

There are also indirect ways for states to control big pharma and steer them to accept more responsibility for human rights. As an instance, it is suggested to mandate a certain level of transparency for pricing and its details, so that everyone will know if the price is logical in comparison to the costs. Although it seems to be an applicable effective approach, it has its own shortages. First of all, it can harm the market and competition since it reveals companies secrets and can be abused by other companies. Also, it can be misinterpreted by people and the media, since nobody knows which margin of interest is fair enough for the company, particularly by considering next projects and future investment programs. Similar suggestion may be made for disclosure of exact amount of donations and also the tax benefit resulted from this donation. In this way the company clearly shows their social contribution and the amount of effort they make to increase accessibility. This method might be more applicable; it is less problematic and makes more sense to public opinion.

All in all, while these top-down approaches might be helpful, the legal complexities have resulted in a long lasting pause in the current situation.

## Bottom-up

The other group of actions are known as bottom-up, which means actions that are taken by society (as individuals or NGOs) to control and/or affect pharmaceutical companies.

The best known- and probably most effective- project in this area is “Access to Medicine Index”. This index was innovated and introduced in 2008 by Bill & Melinda Gates Foundation and measures the amount of activities of biggest pharmaceutical manufacturers in means of increasing access to essential medicine all around the world [21]. In this index 7 fields of activities have been considered with different weights: “Pricing, manufacturing and distributing” with 25 %, “research and development” with 20 %, “Patent and licencing” with 15 % and “general access to medicine management”, “market influence”, “capacity building”, “product donations” each with 10 % share of scores. Each of these fields are assessed based on 4 main criteria: commitment, transparency, performance and innovation. As an instance, Gilead Science Inc. had the highest score in the two fields of “pricing” and “patents” in recent years. This is probably because of increasing access to its new anti-viral medicine sofosbovir (Sovaldi) which has been provided 99 % off price for developing countries [22]. Although this medicine is provided with about $1200 per pill in US [23], Gilead allowed generic licencing in developing countries so that the medicine can be accessible in very low price and save lives of millions of people with hepatitis C, as well as its benefit from the market. Other examples also can be seen in the index report which clearly shows the improvements and lacks of the companies’ policies.

At first glance it might be a simple index like thousands of other scientific measures. However, looking closer at the index one can notice that it is effectively encouraging big pharma to increase the access to their products in their own way and without any legislative obligation. Hogerzeil et al. showed that average score of these big pharmaceutical companies increased since 2010 to 2012 and we can be hopeful about continue of this increasing trend [24]. The reasons that may explain this positive effect, first is the attempts of companies to improve their image in the public opinion, which might be a promotion for their products and attract direct costumers. And second, the effect of improving public image on share values and convincing more shareholders to invest on the company. However, this encouraging effect is still a theory and not proven yet, particularly in long term. By looking at the index for 2014 we can see a decreasing trend for most of the companies since 2012. It seems that even if it is effective, it cannot be a guarantee for improvement of access to medicine overtime.

In addition, this measure potentially can be used as a precise definition for crimes, and to make legal actions for controlling pharmaceutical companies. As a broad example, we can imagine a law that mandates having a minimum score of Access to Medicine Index, and companies which get lower score will be penalized. In this way Access to Medicine Score is not just an honor which can be neglected by some companies or in some situations, but pharmaceutical companies have to try for it. This can be an answer to our question about “definition of crime” which mentioned before.

Other bottom-up approaches are also using the same trick. “Naming and shaming” by NGOs and world organizations can be an effective way, using “punishment” instead of encouragement. In this way, campaigns and movements can be used to openly blame the companies which explicitly avoid helping for improving access to medicine, so that the public opinion and media stream may make a pressure to these companies for accepting their responsibilities.

## Current legal challenges and future efforts to make

Although it is 2015 and at least main parts of this problem were supposed to be solved by now, there is still a big capacity to change the situation. Having only some ethical guidelines and declarations after all these years shows a hanging situation and can be a clue that we need a break in the current framework to make a new paradigm. This new paradigm might be a different point of view in assumptions and philosophical theories that are being used as basis of world legal systems.

As an example, currently human rights are still interpreted from its conventional point of view. Human rights were initially established to protect mainly ‘human’ from the ‘state’, and majority of laws gave the originality to individuals and their freedom. But during these decades there has been a shift in the society to where the state does not have its previous major role in many areas, and non-state actors like pharmaceutical companies have started to play a more important role, which means there is a need to protect individuals from other ‘individuals’ who are free to make inequitable conditions- such as inequity in medicine production or pricing. Despite all these shortages and complications, there are no legal obligations for the private sector to obey human rights, i.e., the human rights are not ‘legally binding’ directly to pharmaceutical companies. The new legal framework is to find new ways to guarantee the proper contribution of these companies in human rights.

As a legal suggestion, an international effort can be made to oblige big pharma to sign the human rights treaties. By this mean, the enquiry of pharmaceutical companies and the assessment of their practice will be legally possible by international organizations. The first question will be if pharmaceutical companies are legally allowed to sign the treaty. One may refer to International Covenant on Economic Social and Cultural Rights (ICESR) article 8, where trade unions are known as legal persons in this law to have the right, and by definition, we may conclude that legal persons may be addressed to legal obligations as well as legal rights. Following this interpretation, even if pharmaceutical companies are not able to sign the covenants themselves, their professional union or association- either international or domestic- can be obliged to sign treaties and accept the commitments to provide affordable and accessible medicine for all disease categories.

Moreover, one of the major problems with current paradigm is the lack of incentives for states to make these new regulations, either because they do not see the access to medicine as their own problem, or because they are taking the advantage of the taxes coming from these pharmaceuticals, and restricting them is equal to losing part of this huge tax. Hence, governments might not be much interested in these types of regulations even though it is against their commitments to human rights covenant. Drawing on this point of view, world authorities may be able to establish new laws not only for controlling the trade and accountability of manufacturers, but also for more contribution of states. If member states be known as main responsible (instead of pharmaceutical companies) they will have enough incentives to find new ways to make pharmaceutical companies to co-operate or to compensate their share of contribution.

Legitimacy of TRIPS-plus agreements also can be criticized as a barrier for realization of human rights. According to Article 41 the Vienna Convention on the Law of Treaties, although having unilateral or multilateral agreements or modifications within members is allowed, it should be only in a way which does not affect the enjoyment of other members of treaty from the enjoyment of their rights as defined in the treaty. In our case of pharmaceutical companies, when United States- as one of the major owners of pharmaceutical companies- makes TRIPS-plus agreements and expand the patent duration in several countries- while some of them own big generic producers, and many of them are big generic consumers- it can be against the enjoyment of other member states, either those which want to buy generic products, or those that want to export generic products to subjected developing countries. Hence these agreements are not just restricting for the countries signing them, but also for many other TRIPS members.

In addition to these flaws of TRIPS-plus agreement, it can be also criticized in a human rights point of view that suggestion of such an agreement to developing countries- while it is predictable that the country will have big problems in purchase of medicine- is against human rights.

To sum up, the most important actions to take are making pharmaceutical unions or associations responsible by signing human right treaties, increasing the responsibility and contribution of member states and making TRIPS-plus agreements less effective.

## Conclusion

Health is a basic human right and access to medicine is a basic tool to ensure health. This right and its tools are facing major issues in the world. Pharmaceutical companies play a substantial role in increasing the access to medicines in order to guarantee health.

Till now and despite all shortages and difficulties, remarkable efforts have been made. Major pharmaceutical companies are helping billions of dollars by donating medicine to poor population or patients with neglected disease. WHO and human rights committee of United Nations also make several heads-up every year and seek new ways to improve the situation of access to medicine in co-operation with NGOs and governmental organizations. Also states have tried to create a better circumstance for increasing the availability and affordability of medicine. However, there is still a substantial need for improvements, as well as the notable potential for making changes.

More realistic accountability of different parties- including both states and big pharmaceutical companies- is the main necessity in this way. Without this accountability, there will be no real and long term change in the situation and every step would work as a temporary painkiller, but not as a cure.

Also it should be considered that this accountability cannot be realized automatically or through ethical advice, but needs serious legal acts in terms of defining crimes, binding state and non-state parties to increase their cooperation and to make a safe way for access to generic medicine in developing countries by restricting TRIPS and TRIPS-plus agreements. With all these efforts we can be hopeful that delayed goals for increasing health and decreasing inequity will be achieved.
